# Community capacity for prevention and health promotion: a scoping review on underlying domains and assessment methods

**DOI:** 10.1186/s13643-023-02314-1

**Published:** 2023-08-22

**Authors:** Vera Birgel, Lea Decker, Dominik Röding, Ulla Walter

**Affiliations:** Hannover Medical School, Institute for Epidemiology, Social Medicine and Health System Research, Carl-Neuberg-Str. 1, Hannover, 30625 Germany

**Keywords:** Community capacity, Community capacity assessment, Prevention and health promotion, Community intervention, Scoping review

## Abstract

**Background:**

Building community capacity is an essential health promotion approach, which refers to the characteristics of communities that affect their ability to identify and address social and public health problems. Despite general agreement about certain capacity domains and frameworks, there is no comprehensive and consistent assessment of community capacity. Therefore, the aim of this scoping review is to identify the domains and methods used to assess community capacity related to community-based prevention and health promotion.

**Methods:**

A scoping search was performed on 06/02/2022 via PubMed, Web of Science, and Science Direct, with supplemental searches via Google Scholar. The review included studies published in English from 1990 to 2022 that explicitly described how community capacity was assessed in health promotion and prevention interventions. Furthermore, studies had to meet at least two of the three following criteria for capacity assessment: a theoretical foundation, a participatory approach, or a field test of the assessment tool.

**Results:**

From 4779 records, 38 studies were included after applying exclusion criteria. Nineteen studies used mixed, eleven qualitative and eight quantitative methods to assess community capacity. The various domains used to assess community capacity were identified and reassembled into nine comprehensive domains: *community participation*, *knowledge and skills*, *resources, leadership*, *community power*, *sense of community*, *collaboration*, *critical awareness and problem-solving*, and *community structure*. The review also identified four sub-domains, which include *commitment*, *communication*, *shared values and goals*, and *sustainability*.

**Discussion:**

This scoping review provides an overview of the domains and methods used to assess community capacity, which can facilitate the development of a comprehensive approach to capacity assessment in future research.

**Supplementary Information:**

The online version contains supplementary material available at 10.1186/s13643-023-02314-1.

## Background

The importance of supporting the involvement of communities in efforts to enhance their health is well recognized since it became evident that traditional, top-down health directives often resulted in little to no health benefits [1]. In contrast, participatory approaches have proven effective, resulting in increased interest in the theoretical and practical components of these approaches [2, 3]. However, developing and implementing effective health promotion and prevention programs require careful consideration of contextual factors related to both objective and subjective aspects of a community’s social and built environments [4–10]. While objective measures such as demographic and healthcare data can be obtained from administrative sources like census data, capturing important subjective factors such as community cohesion, trust, and leadership can be challenging. Understanding these subjective factors is crucial to accurately assessing a community’s capacity and performance in implementing and maintaining community-based health promotion and prevention programs [11, 12].

A promising and complex way of approaching contextual factors of communities is by assessing their capacities. Community capacity refers to a set of dynamic characteristics of communities [13] and can be defined as the interplay of human capital, organizational resources, and social capital used to solve collective problems and improve the well-being of a community [14]. However, there is a lack of consensus on measuring capacity in communities [15, 16]. To date, community capacity is not well understood and has been associated with various meanings, frameworks, and assessment tools [17–19]. It has proven to be challenging to measure, and its value is often rendered invisible or underestimated. For this reason, there have been few attempts to develop a theoretical approach to identify a relationship between community capacity and positive health outcomes [20].

It is essential to note that community capacity is not a static concept. Instead, it comprises two essential components, existing capacity, and the need for further capacity building over time [21]. Existing community capacity is an important foundation that supports further capacity building by developing existing resources, creating effective community organizations, and institutions [21]. Therefore, a comprehensive understanding of community capacity requires a clear appreciation of these two essential components as they are both critical in achieving positive health outcomes.

Previous reviews have examined the tools and methods used to assess *community capacity building* [15, 22]. However, these reviews have primarily focused on public health interventions in general and have not specifically delved into the assessment of community capacity. For instance, a systematic review conducted by Liberato et al. (2011), which covered literature up to 2010, identified a consistent set of domains used to describe and assess capacity building in community interventions [15]. Similarly, van Herwerden and colleagues (2019) conducted a systematic review that encompassed literature up until 2017 and identified methods employed to measure capacity building processes in public health community interventions [22]. Other reviews have described specific elements of community capacity, primarily specific capacity frameworks with domains [23, 24].

Although these reviews have provided valuable insights into community capacity building, they did not specifically focus on the assessment of community capacity within the context of prevention and health promotion interventions. Studies have indicated that communities with higher levels of community capacity are more successful in implementing community-based health promotion and prevention programs [25–28]. Thus, it is essential to assess community capacity prior to implementing interventions to identify any existing deficiencies that can be addressed through capacity building efforts. Moreover, to ensure that community interventions can build on existing capacities, it is essential to focus not only on capacity building processes but also on community capacity itself. Therefore, the objective of this scoping review is to provide a comprehensive overview of the current literature on the assessment of community capacity in the field of prevention and health promotion. More specifically, the review focused on domains and methods used to assess community capacity in the context of prevention and health promotion interventions.

The review aims to address the following questions:

1. What are the different domains used to assess community capacity in the context of prevention and health promotion interventions?
2. What are the different methods used to assess community capacity in the context of prevention and health promotion interventions?

## Methods

This study used a scoping review methodology developed by Arksey and O’Malley [29] with the modifications recommended by Levac et al. [30]. Our five-stage scoping review model included the following: (i) identifying the research question, (ii) identifying relevant studies, (iii) selecting studies, (iv) charting data, and (v) summarizing and reporting the results. The PRISMA Extension for Scoping Reviews (PRISMA-ScR) checklist [31] was followed for reporting purposes.

### Search strategy

A scoping search was conducted on 06/02/2022, using three databases (MEDLINE, Web of Science, ScienceDirect), supplemented by Google Scholar searches, using the search terms outlined in Table 1. A list of search strategies for each database can be found in Supplementary file 2. In addition, the reference lists of included studies and relevant previous reviews were screened for additional studies.

**Table 1:** Database search terms

Capacity search terms AND	Prevention and health promotion search terms AND	Assessment search terms
Community capacity or Capacity building or Community engagement or Community participation or Project management	Prevention or Health promotion or Community intervention	Evaluat* or Assess* or Measure*

*denotes a truncation command used to capture all possible suffix variations of the root word

### Inclusion and exclusion criteria

Studies published in English between 1990 and 2022 were included if they reported original research or review articles (including meta-analyses and systematic reviews) focused on assessing community capacity related to community-based health promotion or prevention. Community-based prevention and health promotion were defined as a strategic set of activities focusing on enhancing the community’s ability to address and prevent the root causes of diseases and to improve their health outcomes [32]. No restrictions were made regarding study type or country of origin.

The reasons for selecting 1990 as the threshold for inclusion of literature are based on three factors. First, the start of a new era in community development with new policies and initiatives aimed at promoting community capacity building [33, 34]. Second, many of the foundational theories and concepts related to community capacity building (e.g., social capital) were developed during the 1990s, making this an important period for the field [35]. Third, the concept of community capacity building itself also gained prominence during the 1990s, with scholars and practitioners beginning to focus on building the capacity of communities to address their own needs and priorities [36].

Assuming that the inclusion of the respective community in the design or testing of the assessment tool contributes to a higher quality of the capacity assessment [37], studies had to meet at least two of the three following criteria:

- The underlying domains of community capacity were theoretically founded.
- The assessment tools were developed using a participatory approach.
- The capacity assessment tools were tested in the field.

Studies were excluded if they (a) did not assess community capacity, (b) were not related to prevention or health promotion, or (c) measured processes of capacity building without specifying areas of community capacity. Multiple reports arising from the same study were treated as a single study.

### Screening and selection of studies

Two reviewers (V. B., L. D.) independently screened the articles by applying the eligibility criteria. In the first stage of screening, titles and abstracts were scanned for any information on potential community capacity assessments (e.g., an aim of the study was to increase community capacity). Authors checked for consensus on their decision to include or exclude studies before moving on to the next stage. In the second stage, full texts were screened for the three inclusion criteria listed above. All conflicts generated throughout the screening stages between the two reviewers were discussed until a consensus was reached.

### Data extraction

A data extraction template was developed to answer the research question. Two authors performed data extraction independently (V. B., L. D.), with any disagreement resolved by conferring with a third author (D. R.). Information extracted from each study included the following: (a) author and year, (b) study aims, (c) study design and methods, (d) sample characteristics, (e) capacity domains, (f) underlying theoretical frameworks, and (g) assessment tools. Due to heterogeneous characteristics of included studies, commonalities and differences between underlying domains, capacity assessment tools and frameworks, and methods were identified and described narratively. Results of the data extractions are presented in Supplementary Tables 1–4 (see Additional file 1: Supplementary table 1).

### Data synthesis

Data extracted from the included studies were synthesized descriptively. The included studies were summarized based on the study aims, design, sample characteristics, capacity domains, underlying theoretical frameworks, and assessment tools used. Commonalities and differences between the studies were identified and described narratively. Due to the heterogeneity of the included studies, a meta-analysis was not feasible. Instead, the included studies were synthesized descriptively, and key findings are summarized in this review.

### Quality assessment

While quality assessment is not mandatory in scoping reviews, we decided to conduct a quality assessment of the included studies in this review to enhance the credibility and usefulness of the review findings [30, 31, 38]. In this scoping review, the Mixed-Methods Appraisal Tool (version 2018) (MMAT) was used to assess the quality of the included studies. The tool is a validated and reliable instrument for assessing the methodological quality of different types of studies [39]. By using the MMAT, it allowed us to use one quality assessment tool to review all studies. We used the MMAT to review study quality, but we did not exclude any study based on their quality. Two authors (V. B., L. D.) completed this independently in consultation with a third author (D. R.).

## Results

**Fig. 1:**
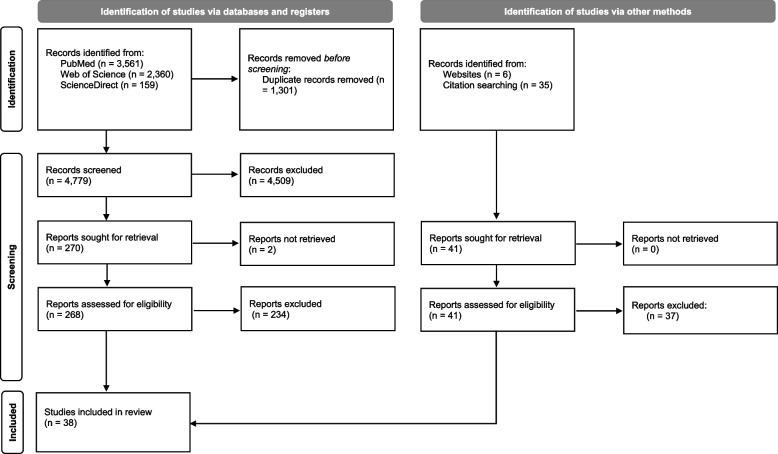
PRISMA 2020 flow diagram for the scoping review process

The search strategy yielded 4779 results after removing duplicates. Figure 1 presents a PRISMA flow diagram from search to the final inclusion of the studies according to Page et al. (2021) [40].

Three percent of the identified studies were published before 2001, 33% between 2001 and 2011, and 65% between 2012 and 2022, indicating an increasing interest in community capacity. After exclusion criteria were applied, 38 studies were included in the final analysis [16, 25, 26, 41–74]. In 28 studies, capacity was assessed in one of six developed countries (USA, Canada, Australia, Germany, New Zealand, Spain). Ten studies captured community capacity in one of seven developing countries (South Korea, Ghana, Guinea, Thailand, Zambia, Fiji, South Africa). Further details on the characteristics of included studies can be found in Supplementary Table 1 (see Additional file 1: Appendix 1).

### Quality assessment

In the quality assessment process, the studies were evaluated using the Mixed Method Assessment Tool. This tool encompasses seven quality criteria for each study type. For detailed information regarding the quality assessment, please refer to Supplementary file 1, Table 4.

Among the qualitative studies, nine out of eleven studies [44, 50, 54, 55, 62, 64, 68–71] adhered to all quality criteria, demonstrating clear research questions, appropriate study designs, adequate data collection methods, interpretation supported by data, and coherence within the study. However, one study had an unclear risk of bias in three criteria [65].

For the quantitative non-randomized studies, one out of two studies [25] met all criteria, including clear research questions, representative participants, complete outcome data, and proper intervention administration. However, the other study had an unclear risk of bias regarding participant representativeness [47].

Among the six quantitative descriptive studies, only one [56] fulfilled all quality criteria, including clear research questions, relevant sampling strategies, representative sample, appropriate measurements, low risk of nonresponse bias, and proper statistical analysis. However, the remaining studies [51, 52, 57, 58, 66] did not meet one to two criteria, such as unclear sampling strategies, lack of participant representativeness, high risk of nonresponse bias, and inadequate measurements or statistical analysis.

Regarding the mixed-methods studies, eleven out of the nineteen included studies [16, 42, 43, 46, 53, 61, 63, 67, 72, 74, 75] fulfilled all quality criteria, including clear research questions, integration of components, adequate interpretation of results, and addressing divergences and inconsistencies between results. However, six studies had an unclear risk of bias in one to two criteria [41, 45, 48, 59, 60, 73], and one study did not adequately address divergences and inconsistencies between quantitative and qualitative results [26].

Overall, 23 studies (61%) across all study types adhered to all quality criteria, while 15 studies (39%) had some degree of risk of bias or unclear bias for one to three criteria. Based on the results, it can be assumed that no study had a high risk of bias.

### Capacity domains

In the included studies, 80 domains were used to assess community capacity. One study did not use capacity domains to assess capacity, instead described community capacity and capacity building processes qualitatively [54]. Many commonalities and overlaps exist among these 80 domains. Thus, in the context of this review, domains were reassembled into a comprehensive set of nine domains with four sub-domains (see Additional file 1: Appendix, Supplementary Table 2). This involved regrouping domains that were named differently but were based on similar definitions, as well as reassembling domains that represented more than one domain or overlapped with others. The following section provides insight into this process.

Often, domains were named differently in the included studies, although they were based on the same or similar definitions. For instance, *collaboration* was referred to as *social and interorganizational networks* [43] and *networking and cooperation* [66]. In some cases, domains represented more than one domain and were reassembled into two domains. For example, *role of outside agents/power* was reassembled into *resources* and *community power* [62]. A *goal-directed network* was also found to represent two domains, namely *collaboration* and *shared values and goals* [52]. Other domains were found to overlap and were reassembled into one domain. As for instance, a *sense of place* and *community attitudes* identified by Hargreaves et al. (2020) were both reassembled into *sense of community*. *Collaborations* and *partnerships* were both grouped into *collaboration* [74]. Supplementary Table 3 (see Additional file 1: Appendix 1) provides definitions for each of these domains, drawing on the domain features and characteristics described in the studies.

The community capacity domain *resources* was mentioned most frequently (*n* = 33) in the included studies, referring to the attainment of funds, personal resources, and infrastructure. Thirty-two studies considered the domain *leadership* for capacity assessment. It was described as essential in motivating communities to work towards a goal and overcome conflicts. *Participation* featured strongly in 25 studies. It was often viewed as complementary to leadership by addressing community concerns through collective action. The domain *collaboration* was used in 25 studies. It was described as essential in terms of horizontal and vertical linkages as well as collaborations within and across communities. A *sense of community* was identified in 23 studies and comprised of connectedness among community members and a high level of concern for community issues. Its sub-domain *commitment* (*n* = 5) described members’ perceived responsibility for improving the community. Finally, 20 authors underlined the importance of *critical awareness and problem-solving* in the community. These domains were represented in more than half the models or frameworks of the reviewed papers. The remaining three domains were less frequent but were still identified often, with some having sub-domains. *Knowledge and skills* of the project team and community were described as necessary for community capacity in 18 studies. *Community power* to implement, develop, and sustain programs and create change was recognized as essential for community capacity in 12 studies. Twelve studies referred to the importance of *community structure*, with this domain having two sub-domains — the presence of *shared values and goals* (*n* = 6) and *sustainability* (*n* = 6). In both the *community structure* and *shared values and goals* domain, a shared value system was emphasized that supports inclusion of a variety of groups as well as the development of shared vision. Besides these social structures, community structure also refers to economic, political, and organizational structures. Both *community structure* and *sustainability* refer to program management and organizational structures. While community structure also emphasizes other structural features, such as community composition and values within a community, the sustainability domain refers to the specific component of community structure that emphasizes the need for necessary functional structures for program sustainability.

**Fig. 2:**
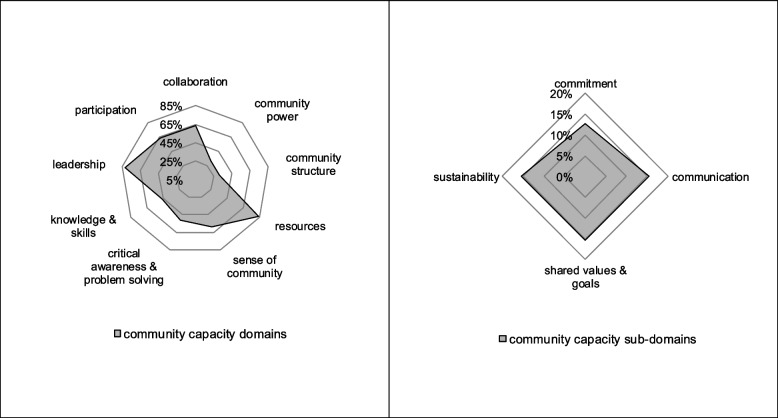
Frequency of domains and sub-domains used to operationalize community capacity

Supplementary Table 4 (see Additional file 1: Appendix 1) shows how the original domains of the included studies were regrouped into nine domains with four sub-domains. Figure 2 illustrates the reassembled domains and their frequency in the included studies.

### Capacity assessment methods

#### Tools and frameworks

This scoping review identified 13 established community capacity tools and 7 theoretical frameworks that were used or adapted for assessing capacity in the 38 included studies. The *tools* referred to well-known validated assessment tools, while *theoretical frameworks* were understood as established models used to describe community capacity and its underlying characteristics, thus providing the basis for the conceptualization of assessment tools. Table 2 provides an overview of established community capacity tools (*n* = 13) and theoretical frameworks (*n* = 7) that were used or adapted for assessing capacity in the 38 included studies.

**Table 2:** Tools and frameworks used to assess capacity in included studies (*n* = 38)

Community capacity tools		*n*
Community capacity questionnaire	Lempa et al. (2008) [61]	3
Community capacity building tool	Public Health Agency of Canada 2013 [76]	3
Community capacity index	Bush et al. (2002) [77]	3
Lovell’s community capacity tool	Lovell et al. (2015) [63]	1
Semi-standardized monitoring instrument	Sauter et al. (2020) [78]	1
Getting to outcomes tool	Chinman et al. (2004) [79]	1
Community readiness to change tool	Oetting et al. (1995) [80]	1
Community capacity theoretical frameworks		
Goodman’s model of community capacity	Goodman et al. (1998) [27]	5
NSWH capacity building framework	NSW Health Department 2001 [81]	2
Freudenberg’s community capacity framework	Freudenberg et al. (2004) [82]	2
Chaskin’s capacity building framework	Chaskin et al. (2001) [14]	2
Norton’s community capacity framework	Norton et al. (2009) [13]	1
Wendel’s model of community capacity	Wendel et al. (2009) [83]	1
Labonte and laverack’s community capacity framework	Labonte & Laverack (2001) [84]	1

Thirteen studies were based on one of seven established community capacity measurement tools and questionnaires [61, 63, 76, 78–80, 85]. The Community Capacity Questionnaire by Lempa et al. (2008) was used in three studies [43, 45, 65]. Lempa et al. (2008) quantitative measure of capacity is based on findings from a multiple-case study for instrument construction and testing among 291 initiatives nationwide.

The Community Capacity Building Tool of the Public Health Agency of Canada [76], which was also utilized in three studies [23, 47, 59, 70], is a planning tool to help communities assess, plan, and build capacity in health promotion projects. It is mainly focused on building capacity within community-based programs. Three additional studies utilized the Community Capacity Index [85], which outlines four fundamental dimensions of health promotion capacity: network partnerships, infrastructure, problem-solving capacity, and knowledge transfer. The index allows for three capacity level assessments in each domain. Lovell and colleagues’ tool [63], as well as Sauter and colleagues’ Semi-standardized Monitoring Instrument [78], were each used once in the included studies [59, 62]. In one study, the Getting to Outcomes Tool developed by Chinman [79] was utilized. One study used a community readiness tool to assess community capacity [25]. The Community Readiness to Change tool measures the capacity of a community’s readiness for action [80].

Twelve studies [42, 46, 48, 50, 56, 64, 65, 67–69, 71, 74] based their measurements on community capacity domains from seven established theoretical frameworks [27, 81–84, 86–89]. The most common framework was Goodman and colleagues’ model of community capacity [27], used to operationalize capacity in five studies [45, 48, 50, 64, 65]. Goodman et al. (1998) identified dimensions of community capacity as leadership, citizen participation, skills, resources, social and organizational networks, sense of community, understanding of community history, community power, values, and critical reflection. Another frequently applied framework was the New South Wales Health Capacity Building Framework [81], used to assess capacity in two studies [26, 74]. The framework provides a guide for enhancing the capability of communities to improve health. It emphasizes five key action areas in capacity building within programs, i.e., organizational change, workforce development, resource allocation, partnerships, and leadership. Freudenberg’s [82] and Chaskin et al. [86] frameworks were used in two studies. The frameworks developed by Norton et al. [89], Wendel et al. [83], and Labonte and Laverack [84] were utilized in one study each.

Seventeen studies used or developed their own measuring tools [16, 51–53, 55, 58, 61, 63, 66, 67, 72, 75] or a new theoretical approach to community capacity [44, 49, 54, 57]. These are detailed in Supplementary Table 1 (see Additional file 1: Appendix 1).

#### Study designs

A variety of capacity assessment methods were used in the included studies (see Supplementary Table 1, Additional file 1: Appendix 1). Most studies (*n* = 19) used mixed methods, while 11 studies used qualitative methods, and eight studies used quantitative methods. Of the studies that used quantitative methods, six studies used a descriptive study design [51, 56–59, 66], and two studies used a cluster non-randomized approach [25, 47].

As there were no restrictions on the time frame of the studies included, community capacity assessment timelines were ambiguous. Twenty-three studies assessed community capacity cross-sectionally without repeated measurements and provided a narrative description of community capacity or capacity building. Fifteen studies also measured the development of community capacity and community capacity building processes over a period ranging from 12 weeks [47] to 10 years [66]. Most studies used a prospective study design. Six studies [26, 45, 48–50, 56] assessed community capacity retrospectively, using data collected from previous community capacity building projects or official data sources.

## Discussion

Community capacity plays a vital role in promoting successful prevention and health promotion interventions. The capacity of a community to mobilize resources, build partnerships, and implement effective interventions is crucial in addressing public health challenges. Despite the critical role that community capacity plays in promoting effective prevention and health promotion interventions, it is not systematically and consistently assessed in community interventions due to its complexity.

To address this gap, a scoping review was conducted to provide a comprehensive overview of the current literature on the assessment of community capacity in the field of prevention and health promotion. Specifically, the review focused on (I) domains and (II) methods used to assess community capacity in the context of prevention and health promotion interventions.

Regarding the first research question, the scoping review identified key domains of community capacity, including community participation, knowledge and skills, resources, leadership, community power, sense of community, collaboration, critical awareness and problem-solving, and community structure. While these domains demonstrated relevance in assessing community capacity across diverse settings and populations, there was less agreement on the domains of community power, critical awareness and problem-solving, and community structure. The review also identified relevant sub-domains such as commitment, communication, shared values and goals, and sustainability.

However, the scoping review found that there were differences in the definition and interpretation of these domains and sub-domains, with some studies considering them comprehensively, while others focused on specific components. To address these differences, it is essential to establish an agreed-upon approach to defining and planning capacity processes, which could be achieved through the development of standard definitions, frameworks, and guidelines for community capacity assessment.

The findings of this review are consistent with previous literature on the core domains for assessing capacity. The literature review by Liberato et al. [15] supports our work by identifying similar constructs (i.e., leadership, resource mobilization, participatory decision-making, partnership/linking/networking, sense of community, learning opportunities and skills development, and development pathway) as the most widely accepted domains of community capacity building.

In addressing the second research question, the review identified a total of 14 frameworks and tools that were utilized for the assessment of community capacity. Among these, the Community Capacity Questionnaire and the Community Capacity Building Tool developed by the Public Health Agency of Canada were the most frequently used tools.

Given that mainly mixed-methods designs were applied in the included studies, it can be assumed that triangulation of qualitative and quantitative methods is particularly useful for capturing community capacity. The inclusion of qualitative methods might add more depth and understanding to capacity assessments than quantitative capacity assessments alone. Qualitative methods allow for a deeper exploration of the community’s experiences, perceptions, and attitudes towards the assessed domains of community capacity. This approach enables researchers to capture the complexity of community capacity and the context in which it occurs. Additionally, qualitative methods can provide an opportunity to identify the factors that promote or hinder the development of community capacity.

The community capacity domains identified in this review have been suggested to be associated with the success of community interventions by several studies. For instance, the presence of leadership has been recognized as crucial for coordinating and developing community programs and evidence-based program selection [90, 91]. Additionally, a sense of community has been identified as an important contextual factor associated with health behavior [92]. Previous studies also found that communities that collaborated to address health problems were more likely to achieve positive health behaviors [60, 72]. Therefore, it is reasonable to suggest that community capacity plays a vital role in promoting successful prevention and health promotion interventions.

To promote more sustainable health outcomes, a strength-based approach that incorporates local experience and knowledge into intervention planning, implementation, and evaluation should rely on community capacity building processes and capture community capacity at the starting point [54]. Given the critical role of community capacity in promoting effective prevention and health promotion interventions, there is a need to continue developing and refining methods to assess community capacity [5, 6].

A comprehensive assessment of community capacity enables interventions to gain insights into a community’s strengths and weaknesses, allowing for the customization of strategies accordingly. By targeting the specific domains of community capacity that are most relevant and addressing inhibiting factors, interventions can enhance their effectiveness in promoting health and well-being.

### Strengths and limitations

This scoping review provides a comprehensive overview of the domains, tools, frameworks, and methods used to assess community capacity in the field of prevention and health promotion. While building on previous reviews [15, 22], this review captures *community capacity* as a means for interventions rather than *capacity building*. It provides a broad range of tools, frameworks, and methods that can be used by researchers and practitioners. The review methodology was rigorous, including independent screening, quality assessment, and synthesis conducted by multiple researchers.

By reassembling the 80 domains used to assess capacity in the included studies, a comprehensive compilation of relevant capacity domains has been provided. This compilation may promote a consistent assessment of community capacity in future research, thus contributing to further insights into the relationship between community capacity and the success of prevention and health promotion interventions. Still, the authors wish to acknowledge that a compilation of the domains may lead to a less precise or extensive picture of community capacity than a presentation of all 80 domains.

One limitation of this review is that it was beyond the scope of the study to assess the impact of community capacity on public health interventions in communities. However, by identifying the domains, tools, and methods used to assess community capacity, this review lays the groundwork for future research to explore this relationship.

Another limitation is the overlap between community capacity and community capacity building constructs, which made it difficult to distinguish between the constructs and apply inclusion and exclusion criteria. Additionally, the review was limited to English-language studies, which could have resulted in the exclusion of important literature in other languages. The exclusion of gray literature may also mean that some relevant studies were missed. However, the references and citations of all included studies were screened, and a limited search of Google Scholar was conducted to identify potentially relevant grey literature. Furthermore, the review did not assess the validity of the identified tools, as it was beyond the scope of this scoping review. However, it is essential to emphasize the importance of evaluating the validity of community capacity assessment tools in future studies. This would involve examining the reliability, accuracy, and consistency of the tools in assessing community capacity across different contexts and populations. Future studies should aim to evaluate the validity of these tools, consider the interplay of various domains, and contrast the domains and tools based on the type or purpose of interventions.

Additionally, no differences between different community types were considered in this scoping review. Conducting a full-scale systematic review would provide researchers with the opportunity to thoroughly assess the validity of the identified tools and thoroughly examine potential variations in community capacity assessment approaches and findings across diverse communities. This comprehensive approach would offer more nuanced insights into community capacity and contribute to the development of tailored interventions based on specific community characteristics.

Despite these limitations, this review provides a first-time account of the assessment of community capacity. It adds to previous reviews by focusing on community capacity as a starting point for interventions rather than capacity building. By offering a comprehensive compilation of relevant domains, tools, and methods, this review can facilitate future research aimed at exploring the relationship between community capacity and the success of prevention and health promotion interventions. It may also encourage a future consensus on the nature of community capacity to shape it as a reliable, possibly valid, construct.

### Conclusion

In conclusion, this scoping review provides a comprehensive overview of the assessment of community capacity in the context of prevention and health promotion interventions. By identifying key domains, sub-domains, and tools, this review serves as a valuable resource for future research and practice. Future research should aim to evaluate the validity of community capacity assessment tools, consider the variations in assessment approaches across diverse communities, and explore the impact of community capacity in the success of prevention and health promotion interventions and health outcomes.

### Supplementary Information

**Additional file 1: Appendix 1.** Supplementary tables. **Supplementary Table 1.** Methodology of included studies assessing community capacity. **Supplementary Table 2.** Domains used to assess community capacity in the included studies (*n* = 38). **Supplementary Table 3.** Definitions of the reassembled domains and sub-domains. **Supplementary Table 4.** Original reassembled domains. **Supplementary Table 5.** Quality assessment of included studies.

**Additional file 2: Appendix 2.** Search strategies for each database.

## Data Availability

Not applicable.
