# Supplementary material for: Community capacity for prevention and health promotion: a scoping review on underlying domains and assessment methods
Source: Syst Rev. 2023 Aug 22;12:147. doi: 10.1186/s13643-023-02314-1 (PMC10464111; doi:10.1186/s13643-023-02314-1)
Supplement: Supplementary file 1 — Additional file 1: Appendix 1. Supplementary tables. Supplementary Table 1. Methodology of included studies assessing community capacity. Supplementary Table 2. Domains used to assess community capacity in the included studies (n = 38). Supplementary Table 3. Definitions of the reassembled domains and sub-domains. Supplementary Table 4. Original reassembled domains. Supplementary Table 5. Quality assessment of included studies. [file 13643_2023_2314_MOESM1_ESM.docx]

# Appendix 1

Supplementary Tables

Supplementary table 1 Methodology of included studies assessing community capacity

| Reference | Aim of the study | Study design | Sample characteristics | Community capacity frameworks and tools |
| --- | --- | --- | --- | --- |
| Alfonso et al., 2008 | To evaluate the capacity needed to implement and sustain the VERB Summer Scorecard program in Sarasota County | Mixed methods: case study evaluation of the VSS obesity campaign three years after implementation | Teens (ages 9-13 years, *n* = not mentioned) in Sarasota County, Florida, USA | Wilder Collaboration Factors Inventory (1)  Getting to Outcomes tool (2) |
| Anderson-Lewis et al., 2012 | To utilize community collaboration and capacity building processes to lower blood pressure in an African American population via a walking intervention | Mixed methods: observational study. Baseline description of capacity with annual capacity assessment over a 5-year time frame | African American adults (*n* = 64) in Mississippi, USA | Community Capacity Questionnaire (3) |
| Atinga et al., 2019 | To assess community capacity to participate in Community-Based Health Planning and Service | Mixed methods: descriptive study design based on a previous study | Any adult man or woman aged 18+ (*n* = 476) in Ghana, West Africa | A modification of Chaskin et al. 2001 (4) |
| Baugh Littlejohns et al., 2000 | To design, test, and evaluate a community capacity assessment process within a Healthy Communities Initiative (HCI) | Qualitative: descriptive study design, participatory approach | Elnora, Canada (*n*= not mentioned) | Assessment tool developed by same researchers |
| Brazier et al., 2015 | To explore how intervention exposure and capacity within communities influence women’s use of maternal health services during pregnancy and childbirth | Mixed methods: observational study and longitudinal retrospective study design. 20 intervention villages (2007-2009) and 10 comparison villages with no intervention. Data collected over a 6-week period | African women of child bearing age (*n* = 2,335) in the Republic of Guinea, West Africa | Goodman et al. (1998)  Lempa et al. (2008)  Community Capacity Index by Bush et al. (2002) |
| Brock et al., 2019 | To describe the capacity-building processes of a Community-Academic Advisory Board (CAB) involved with the adaptation, implementation, and evaluation of a childhood obesity treatment program in a medically underserved region | Mixed methods: case study of the childhood obesity treatment program ‚iChoose‘ over a 3-year time frame | Overweight children (*n* = 101) in South central Virginia and North central Carolina, USA | Constructs adapted from Sandoval et al. 2012 (5) |
| Chae et al., 2021 | To develop a community capacity-building program for the wellness of merchants in a traditional marketplace and examine its effectiveness | Quantitative non-randomized: quasi-experimental study using a pre- and post-design with a non-equivalent control group. Baseline measurement and 12 weeks after intervention | Merchants from two traditional markets (*n* = 60) in South Korea | Adapted version of Community Capacity Building Tool of Public Health Agency of Canada (6) |
| de Groot et al., 2010 | To determine if the capacity of the Geelong community, represented by key stakeholder organizations, to support healthy eating and physical activity for young children was increased after Romp & Chomp | Mixed methods: observational study and longitudinal retrospective study over a 5-year time frame (2004-2008) | Caucasian young children (ages 0-5 years, *n* = 12,000) in Geelong, Australia | NSW Health CB Framework (7, 8)  Community Capacity Index (CCI), (9) |
| Downey et al. 2010 | To review practical strategies that Delta Nutrition Intervention Research Initiative (NIRI) undertook to encourage community capacity building | Mixed methods: observational study. Timelines are unclear | African American and Caucasian adults in rural communities in Mississippi (M), Louisiana (L) and Arkansas (A), USA (*n* = 3,440 (M), *n* = 21,000 (L) and *n* = 1,400 (A)) | Chaskin et al. (2001)  Goodman et al. 1998 (10)  Labronte & Laverack 2001 (11)  Jackson et al. (2003) (12) (13)(13)(13)(13)(13)(13)(13)(13)(13)(13)(13) |
| Dressendorfer et al., 2005 | To describe the derivation of a conceptual model of community capacity development through exploring the process of partnership formation and collaboration within communities as conducted a heart health promotion programs | Mixed methods: case study evaluation of the four Alberta Heart Health Demonstration Projects over a 5-year time frame | Four sites of the Alberta Heart Health project were evaluated. Samples included different targets, e.g., students, teachers, parents, and adults in small rural communities (*n* = not mentioned) in USA | Development of a new model |
| Garney et al., 2017 | Using a comparative case study to understand how the theoretical framework ‘community health development’ (CHD) influences community capacity | Qualitative: descriptive study design. Three-year comparative multiple case study | African and Native Americans, Asians/ Pacific Islanders, Hispanics/ Latinos, LGBTQ groups, and low-income residents (*n* = 82) in Washington State, USA | Goodman et al. (1998) (10) |
| Griffin et al., 2005 | To develop a tool to measure community capacity and organizational capacity specific to addressing teen pregnancy prevention | Quantitative: descriptive study design | Key leaders (53% females, 80% > 40 years, *N* = 1,516) in South Carolina, USA | New survey based on the South Carolina Adolescent Pregnancy Prevention Initiative Evaluation Survey (SCAPPIE) |
| Hargreaves et al., 2017 | To develop a survey to measure collective community capacity to address adverse childhood experiences and resilience | Mixed methods: descriptive study design | Adult members of a health organization (*N* = 276) in Washington State, USA | Development of a new survey: ACEs and Resilience Collective Community Capacity (ARC3) survey |
| Hernantes et al., 2022 | To design, implement and evaluate a nurse- led capacity building intervention for intersectoral action for health promotion | Mixed-methods: descriptive study design | Individuals (n=19) representing various sectors at a local government in northern Spain | Development of a new tool (CaPSalGOB) |
| Jackson et al., 2003 | To develop a model of community capacity and explore how one might measure community capacity based on community experience | Qualitative: action research project over a four-year time frame | Adult residents and agency workers from a wide variety of ethnicities, including African, Chinese, Indian, Somalian (*n* = 161) in Toronto, Canada | Development of a new model |
| Jones et al., 2017 | To examine the efficacy of capacity building strategies in community sport organizations, and provide contributions to community capacity theory | Qualitative: case study involving a youth center | Multi-ethnic, low-income neighborhood in a medium-sized city (*n* = not mentioned) in Southeastern USA | Researchers own model |
| Jung, 2012 | To evaluate the relationship of community capacity and health behaviors in a Korean metropolitan area | Quantitative: descriptive research design using retrospective official data sources | Adult men and women (ages >20 years, *n* = 14,228) in 25 districts of Seoul, South Korea | Wendel et al. 2009 (14) |
| Jung et al., 2013 & Jung & Viswanath, 2013 | To evaluate whether community capacity is correlated with the residents’ levels of health and to identify the decisive factor in increasing the community capacity | Quantitative: descriptive research design | Adult men and women (ages >20 years, *n* = 14,228) in 25 districts of Seoul, South Korea | Development of a new model |
| Kegler et al., 2011 | To evaluate the California Healthy Cities and Communities program (CHCC) and assess mediating effects of community capacity outcomes | Quantitative: descriptive study design. Project evaluation over a three-year time frame | Coalition members of 19 healthy cities and communities’ coalitions (*n* = 231) in California, USA | Development of a new tool |
| Kim et al., 2009 | To assess the community capacity building ability of health promotion workers of public health centers and to identify influential factors to the ability | Mixed methods: descriptive study design | Metropolitan City public officers from 16 public health centers *(n* = 43) in Busan, South Korea | New survey based on the Community Capacity Building Tool (6) |
| Kim et al., 2020 | To identify the level of active aging in older adults and the influence of the individual and community levels of community capacity on active aging | Quantitative: descriptive study design | Older adults (*n* = 380) in Seoul, South Korea | Individual level capacity: Community Capacity Instrument (15)  Community level capacity: based on a previous study |
| Lempa et al., 2008 | To describe the development of two capacity measures of local public health initiatives for leaders and non-leaders | Mixed methods: descriptive study design | Respondents representing community initiatives: leaders (*n* = 251), core participants (*n* = 264), peripheral initiative participants (*n* = 187) in USA | Development of a new tool |
| Loss et al., 2020 | To establish stakeholder groups in two communities and to monitor and examine the capacity building processes | Qualitative: descriptive study/ participatory approach. Measurement over a three-year time frame | Two rural communities (Community A: *n* = 27, Community B: *n* = 30) in Bavaria, Germany | Semi-standardized monitoring instrument (16) |
| Lovell et al., 2015, Lovell et al., 2017 | To develop a scale measuring community capacity, to explore predictors of community capacity scores, and to analyze the relationship between community capacity and self-rated health | Mixed methods: descriptive study/ sequential exploratory design | Adults of mostly European and Maori ethnicity (*n* = 306) from four small high-deprivation towns (Mataura, Milton, Winton, Riverton) in New Zealand | Development of a new tool |
| Maclellan-Wright et al., 2007 | To develop valid and reliable scales to track changes in community capacity to address health issues throughout the course of funded projects | Mixed methods: sequential exploratory design. | Think tank participants (*n* = 21), health promotion and community practitioners (*n* = 9) and organizations (*n* = 29) from different regions of Canada | Development of a new tool |
| Merzel et al., 2008 | To explore how members of one community identify and interpret key aspects of their community‘s capacity to limit the availability and use of tobacco products | Qualitative: participatory research approach. | Socioeconomically disadvantaged community of color (77%) with a high percentage (22%) of smoking adults (*N* = not mentioned) in Harlem, New York City, USA | Goodman et al., 1998 (10) |
| Millar et al., 2013 | To determine changes in capacity over a 3-year intervention (2005–2008) in schools and whether greater increases in capacity were associated with greater decreases in obesity | Quantitative non-randomized: cluster controlled pre-post study design in secondary schools over a 3-year time frame. | Children from 49 secondary schools (*N* = not mentioned) in Barwon-South Western (BSW) Victoria, Australia | Community Readiness to Change Tool (CRT) (17) |
| Motley et al., 2013 | To evaluate community capacity among an emerging coalition initiated to address complex regional obesity problems | Qualitative: case study design over a three-year time frame. | Young People of largely European descent, ages 12-18 years (*n* = 12 schools; *n* = 108 interviews), in Geelong, Australia | Goodman et al. 1998 (10)  Lempa et al. 2008 (3)  Gibbon et al. 2002 (18)  Maclellan-Wright et al. (2007) |
| Nickel et al., 2018 | To assess community capacities with a newly developed instrument ‘KEQ’ in a disadvantaged neighborhood. | Quantitative: assessment of community capacities in three surveys over a ten-year time frame | Residents of a disadvantaged neighborhood called Lenzsiedlung (*n* = 40-60 per survey), Germany | Researchers own tool: Capacity building in small areas/ neighbourhoods (KEQ) |
| Oetzel et al., 2011 | To develop a measure of community capacity for American Indian communities | Mixed methods: participatory research approach/ sequential exploratory design | American Indians (*N* = 500) from two southwest tribal communities in USA | Development of a new tool  Goodman et al. 1998 (10)  Norton et al. 2002 (19) |
| Parker et al., 2010 | To identify the enhancement of community capacity to reduce physical and social triggers associated with childhood asthma and factors that facilitated or inhibited the enhancement of community capacity | Qualitative: descriptive study design with a participatory approach to evaluate the CONEH project. | Steering committee members (*n* = 10), CONEH staff (*n* = 5) and key community organization staff members (*n* = 5) in Detroit, USA | Freudenberg’s framework (20) |
| Postma & Ramon, 2016 | To identify health promoters‘ perceptions of housing issues faced by farmworker families in an agricultural community and to strengthen community capacity to promote healthy and affordable housing | Qualitative: descriptive study design. | Mostly Hispanic health promoters (*n* = 6) of farmworker families in an agricultural community in USA | Freudenberg’s framework (20) |
| Raine et al., 2014 | To describe progress in building the capacity of a provincial coalition (Alberta Policy Coalition for Cancer Prevention) to promote chronic disease prevention | Qualitative: descriptive study design/ participatory approach. Pre- (time 1, Feb 2010) and post-survey (time 2, May, 2011) | Members of APCCP (Time 1: *n* = 28, Time 2: *n* = 37) in Canada | Adaptation of the Community Capacity Building Tool of Public Health Agency of Canada |
| Suwanbamrung et al., 2010 | To explore meanings and domains for giving communities the capacity to be successful in dengue prevention and control and to assess the level of community capacity for dengue prevention and control | Mixed methods: multiple studies and descriptive participatory research. | Community leaders (*n* = 60) and non-leaders (*n* = 60) in four high dengue incidence sub-districts of Southern Thailand | Development of new tool: Dengue community-capacity assessment tool (DCCAT) |
| Thompson et al., 2000 | To present the experience in piloting a methodology to assess the extent to which community capacity was built in a rural heart health project | Qualitative: descriptive study with a four-year time frame (1993-1997). | Community members (*n* = varies between 24-40) and agency staff (*n* = 8) in Alberta, Canada | Community capacity web adapted from pentagram used by (21) |
| Underwood et al., 2012 | To develop and validate community capacity indicators, and apply them to the evaluation of a health communication project Zambia (HCPZ) | Mixed Methods: three-phase sequential descriptive design over a five-year time frame (2002-2007). | Field test, as well as survey applied to adults (*n* = 720; *n* = 4,816) in Zambia, Africa | Development of a new tool |
| Van den Broucke et al., 2010 | To describe a project to strengthen the capacity for health promotion in two Provinces in South Africa | Mixed Methods: over a six year time frame. | Community members of Mpumalanga (*n* = 21) and Free State (*n* = 37) in South Africa | Community Capacity Index (CCI); (9) |
| Waqa et al., 2013 | To build the capacity of and empower communities to tackle the issue of unhealthy weight gain in adolescents aged 13–18 years | Mixed methods: quasi-experimental design. Intervention period of 2 years | Student champions (*n* = 200), School Health Committee members (*n* = 100), Steering Committee (*n* = 21) in Suva, Nadi, Sigatoka and Lautoka, Fiji | NSW Health Department 2001 (8) |

Supplementary Table 2 Domains used to assess community capacity in the included studies (n = 38)

|  | Leadership | Resources | Participation | Collaboration | Sense of community | Knowledge & Skills | Community  power | Community structure | Critical awareness & problem-solving | Sub-domains | |
| --- | --- | --- | --- | --- | --- | --- | --- | --- | --- | --- | --- |
| Alfonso, 2008 |  | ✓ | ✓ |  |  | ✓ | ✓ |  |  | Sustainability | |
| Anderson-Lewis, 2012 | ✓ | ✓ |  |  |  |  |  | ✓ |  | Sustainability | |
| Atinga, 2019 | ✓ | ✓ |  | ✓ | ✓ |  |  |  | ✓ | Commitment | |
| Baugh Littlejohns, 2000 | ✓ | ✓ | ✓ |  | ✓ | ✓ |  |  | ✓ | Shared values & goals,  Communication | |
| Brazier, 2015 | ✓ | ✓ | ✓ | ✓ |  |  |  |  |  |  | |
| Brock, 2019 | ✓ | ✓ | ✓ | ✓ | ✓ |  | ✓ |  | ✓ | Commitment, Sustainability, Communication | |
| Chae, 2021 | ✓ | ✓ | ✓ | ✓ | ✓ | ✓ |  | ✓ | ✓ |  | |
| de Groot, 2010 | ✓ | ✓ |  | ✓ |  |  | ✓ | ✓ |  |  | |
| Downey, 2010 | ✓ | ✓ | ✓ |  |  | ✓ |  |  |  |  | |
| Dressendorfer, 2005 | ✓ | ✓ |  |  |  |  |  | ✓ |  |  | |
| Garney, 2017 | ✓ | ✓ | ✓ | ✓ | ✓ | ✓ | ✓ |  | ✓ | Shared values & goals | |
| Griffin, 2005 | ✓ | ✓ | ✓ |  |  |  | ✓ | ✓ |  |  | |
| Hargreaves, 2017 | ✓ | ✓ |  | ✓ |  |  | ✓ |  | ✓ | Shared values & goals,  Commitment, Sustainability, Communication | |
| Hernantes, 2017 |  | ✓ |  |  |  | ✓ |  |  | ✓ | Commitment | |
| Jackson, 2003* |  |  |  |  |  |  |  |  |  |  | |
| Jones, 2017 | ✓ |  | ✓ | ✓ | ✓ | ✓ |  |  |  | Shared values & goals | |
| Jung, 2012 |  | ✓ | ✓ |  | ✓ |  |  |  |  |  | |
| Jung, 2013 |  | ✓ | ✓ | ✓ | ✓ |  |  |  |  |  | |
| Kegler, 2011 |  |  |  | ✓ | ✓ | ✓ |  |  |  |  | |
| Kim, 2009 | ✓ | ✓ | ✓ | ✓ | ✓ | ✓ |  | ✓ | ✓ |  | |
| Kim, 2020 | ✓ | ✓ | ✓ | ✓ | ✓ |  |  |  | ✓ |  | |
| Lempa, 2008** | ✓ | ✓ |  |  |  |  |  |  | ✓ | Commitment, Sustainability, Communication | |
| Loss, 2020 | ✓ | ✓ | ✓ | ✓ |  |  | ✓ | ✓ | ✓ |  | |
| Lovell, 2015 & 2017 | ✓ |  | ✓ | ✓ | ✓ |  |  |  | ✓ |  | |
| Maclellan-Wright, 2007 | ✓ | ✓ | ✓ |  | ✓ | ✓ |  | ✓ |  |  |  |
| Merzel, 2008 | ✓ | ✓ | ✓ | ✓ | ✓ | ✓ | ✓ |  | ✓ | Shared values & goals | |
| Millar, 2013 | ✓ | ✓ |  | ✓ | ✓ | ✓ |  |  |  |  | |
| Motley, 2013 | ✓ |  | ✓ | ✓ | ✓ | ✓ | ✓ | ✓ | ✓ |  | |
| Nickel, 2018 | ✓ | ✓ | ✓ | ✓ |  |  |  |  |  | Communication | |
| Oetzel, 2011 | ✓ |  |  |  | ✓ |  |  | ✓ |  |  | |
| Parker, 2010 | ✓ | ✓ | ✓ | ✓ | ✓ | ✓ | ✓ |  | ✓ |  | |
| Postma & Ramon, 2016 | ✓ | ✓ | ✓ | ✓ | ✓ | ✓ | ✓ |  | ✓ |  | |
| Raine, 2014 | ✓ | ✓ | ✓ | ✓ | ✓ | ✓ |  | ✓ | ✓ |  | |
| Suwanbamrung, 2010 | ✓ | ✓ | ✓ | ✓ | ✓ |  |  |  | ✓ | Sustainability, Communication | |
| Thompson, 2000 | ✓ | ✓ | ✓ |  | ✓ | ✓ |  |  | ✓ | Shared values & goals | |
| Underwood, 2012 | ✓ | ✓ | ✓ |  | ✓ |  |  |  |  |  | |
| Van den Broucke, 2010 |  | ✓ |  | ✓ |  | ✓ |  |  | ✓ |  | |
| Waqa, 2013 | ✓ | ✓ |  | ✓ |  |  | ✓ | ✓ |  |  | |

*Overall indicators for community capacity are described, as perceived by community members. No individual domains.

**Different community capacity domains for leaders and non leaders are described. Collaboration and continuing sustainability is only described for leaders.

**Supplementary table 3** Definitions of the reassembled domains and sub-domains

| Domain | | | Sub-domains | Definitions |
| --- | --- | --- | --- | --- |
| Community participation | |  | | community members’ involvement in community matters (7, 22–28) |
|  | |  | | joined decision-making 29–31 |
|  | |  | | joined problem-solving (32–34, 28) |
|  |  | | | community funding for local endeavors (35, 24, 15, 36, 37) |
|  |  | | | strong organizational base (37) |
|  |  | | | collective action (38, 26) |
|  |  | | | feeling of empowerment (39, 40) |
|  |  | | | links with others (33) |
| Knowledge and skills |  | | | assessment of community skills (7, 32, 27, 34, 41) |
|  |  | | | development of community skills(40, 42) |
|  |  | | | project team skills (22, 23, 43, 25) |
|  |  | | | community skills (22, 23, 30, 31) |
|  |  | | | organizational skills (7, 32, 27, 34, 41) for   - program delivery and process management; - critical situation management; - resource mobilization; - leadership; - ability to engage in group processes |
|  |  | | | creating conditions to exchange and use information within and between organizations and groups within a community network (44, 45) |
|  |  | | | knowledge about local efforts and their effectiveness, causes of the problems, consequences and impacts on the community (46) |
| Resources |  | | | internal resources (28, 47, 22, 25, 26, 30) |
|  |  | | | external resources (30, 28, 22, 3, 25) |
|  |  | | | funding (7, 48, 3) |
|  |  | | | monetary resources (47, 44, 22, 24, 46, 27, 30, 31, 49) |
|  |  | | | availability of people (7, 48, 44, 32, 35, 38, 27, 30, 31, 49, 46) |
|  |  | | | infrastructure (43, 50, 45, 24, 3, 41) |
|  |  | | | equipment (7, 48, 32, 3) |
|  |  | | | facilities (44, 22, 24, 30, 31) |
|  |  | | | support (7, 48, 47, 34) |
|  |  | | | time (7, 22, 46, 30, 44) |
|  |  | | | resource mobilization (51) |
|  |  | | | access to data sources (52) |
|  |  | | | ability to move project goals forward through the distribution of resources and communication channels (37, 39, 43, 33) |
| Leadership |  | | | connectedness to other leaders (37, 50) |
|  |  | | | connectedness community members (33, 15, 25) |
|  |  | | | provision of direction and structure (37, 39, 26, 51, 28) |
|  |  | | | acknowledging community and individual achievements (22, 23, 30, 51, 31) |
|  |  | | | leaders being role models and treating people fairly (53, 28) |
|  |  | | | fostering the development of new leaders and cultivating leadership opportunities (22, 43, 40, 23, 24, 51) |
|  |  | | | facilitating networks, collaborations and opportunities to participate (22, 50, 32, 23, 30, 28, 54) |
|  |  | | | consensus building (48, 3) |
|  |  | | | leadership commitment (48, 47) |
|  |  | | | clear vision (3) |
|  |  | | | leadership skills and experiences (27) |
|  |  | | | Authority (33) |
|  |  | | | trust in leadership and community standing of leaders (29, 52, 3, 15, 25) |
|  |  | | | support through community members (29, 46, 30) |
|  |  | | | ability to deal with problems (30, 51, 28) |
|  |  | | | training (52) |
|  |  | | | ability to promote processes of change (54) |
| Community Power |  | | | power required to implement, develop and sustain programs to   - create change (7, 39) - achieve process and project outcomes (32, 26) |
|  |  | | | development of plans that reflect the needs of a community (7, 45) |
|  |  | | | sharing power among different members of the community (55, 52) |
| Sense of community |  | | | connectedness among members (47, 37, 40, 38) |
|  |  | | | shared values(47, 44) |
|  |  | | | high level of concern for community issues (37) |
|  |  | | | respecting and valuing the viewpoints of other community members (37, 29, 52, 26, 53) |
|  |  | | | trust (44, 22, 23, 30, 51) |
|  |  | | | sharing information (44, 29, 39, 30, 51) |
|  |  | | | learning from each other’s experiences and best practices (30) |
|  |  | | | awareness of community history to understand and address the root causes of (health) issues (44, 37, 22, 32, 40, 23, 24, 15, 30, 31) |
|  |  | | | positive attitudes towards community and its future; feeling of belonging (56, 32, 35, 38, 24, 15, 46, 27, 31) |
|  | Commitment | | | commitment to and responsibility for improving the community (47, 39) |
|  |  | | | demonstrating commitment to established plans and commitment to action (29, 52, 3) |
| Collaboration |  | | | linkages with others (22, 32, 40, 38, 23, 33, 30, 25) |
|  |  | | | partnership (37, 29, 39, 52, 23, 33, 26, 41) |
|  |  | | | connections (15, 24) |
|  |  | | | social cohesion (38) |
|  |  | | | social capital (42) |
|  |  | | | networks (34, 51) |
|  |  | | | group of organizations or individuals who share interests, information and resources (32, 24, 33) |
|  |  | | | working cooperatively across sectors towards one or more common goals (56, 39, 22, 38, 23, 46, 26, 51) |
|  |  | | | horizontal and vertical linkages among participants and their organizations and other relevant local, regional and national groups (27) |
|  |  | | | factors for successful partnerships   - trust in each other to work together (52, 42, 24, 15) - norms of reciprocity (42, 29) - team-functioning (42, 29) |
|  |  | | | process to which decisions are made and how those decisions keep the project moving forward (39) |
|  | Communication | | | channels of communication are identified and utilized to share information openly and regularly (44, 39, 52, 51) |
|  |  | | | communication with community members to inform about projects and project leaders (3) |
|  |  | | | information provided through online platforms, external publicity, initiatives to other groups (54) |
|  |  | | | degree to which the government listens to the needs of community members (29, 53) |
|  |  | | | aids in identifying and solving problems 24, 15 |
| Critical awareness & problem solving |  | | | ability and capacity to work together to identify and solve problems (critical reflection) (39, 45, 24, 15, 26, 41) |
|  |  | | | problem solving needs assessment (51) |
|  |  | | | translating commitment into action (47) |
|  |  | | | reviewing and strengthening defined action plans (52) |
|  |  | | | ability to analyze successes and failures within a project or community (44, 33, 51) |
|  |  | | | ability to analyze root causes of community health issues (22, 30, 31) |
|  |  | | | ability to apply that knowledge to future change (32, 27, 34, 51) |
| Community structure |  | | | program management 33 |
|  |  | | | organizational structures 57, 48, 45, 33, 26 |
|  |  | | | policy making 50 |
|  |  | | | linking with pre-existing economic, political and social community structures 57, 25, 30 |
|  |  | | | achieving work plans and goals 22 |
|  |  | | | members of the community representing various groups and organizations involved in the program delivery 55 |
|  |  | | | developing functional structures, vision, mission and political will 50 |
|  |  | | | program development and implementation with little or no assistance of the facilitating researchers 33 |
|  |  | | | role of elders, youth and women and the speaking of the native language 53 |
|  | Shared values & goals | | | driving collective action to achieve common goals (32, 44, 31) |
|  |  | | | shared community values supporting democracy, inclusion and social justice (40, 27) |
|  | Sustainability | | | capability to maintain a project (39) |
|  |  | | | personal sustainability (3) |
|  |  | | | belief that a program should be sustainable (7) |
|  |  | | | project continuation via active planning, improving feasibility, seeking additional resources and reflecting efforts made by the community 29, 39, 52, 51 |

Supplementary table 4 Original reassembled domains

| Domain | Sub-domains | Reassembled domains |
| --- | --- | --- |
| Community participation |  | - Community participation (7, 37, 29, 39, 22, 43, 32, 55, 40, 35, 38, 23, 24, 33, 15, 58, 44, 25–27, 34, 30, 51, 31, 54) - Community involvement (55) |
| Knowledge and skills |  | - Knowledge and skills (7, 22, 43, 32, 55, 44, 33, 58, 25, 46, 27, 30, 26, 34, 23, 40, 42) - Knowledge (59) - Skills (59) - Learning opportunities and skill development (40) - Learning culture (40) - Intelligence (31) |
| Resources |  | - Resources (7, 48, 47, 37, 29, 39, 22, 45, 43, 32, 23, 55, 24, 60, 3, 33, 58, 44, 25, 46, 27, 30, 51, 28, 41, 31, 54, 59) - External support (22, 23, 25, 30) - Role of outside agents (33) - Volunteers (35) - Nonprofit organizations (35) - Health care resources (51, 54) |
| Leadership |  | - Leadership (47, 48, 37, 29, 39, 22, 45, 43, 50, 32, 57, 55, 52, 40, 23, 24, 60, 3, 54) - Visibility / recognition (48, 3) |
| Community power |  | - Workforce development (7, 29, 45, 31) - Community power (32, 26, 27) - Power (39, 7, 58, 34) - Community impact (52) - Problem assessment (24, 15) - Role of outside agents/ power (33) - Critical situation management (51) |
| Sense of community |  | - Sense of community (57, 32, 22, 29, 38, 23, 15, 25, 58, 26, 53, 27, 34, 30, 51, 28) - Community satisfaction (35) - Understanding community history (32, 58, 27, 34) - Sense of place (24, 15) - Community attitudes (24, 15, 46) - Setting structures (30) - Trust (38) |
|  | Commitment | - Commitment (47, 29, 39, 52, 3) - Community member commitment (47) - Sectoral commitment (59) - Government’s commitment (59) |
| Collaboration |  | - Social and interorganizational networks (48) - Networking and cooperation (54) - Partnerships (29, 37, 39, 52, 23, 33, 26, 41) - Decision-making (39) - Group roles (39) - Working group (51) - Social and interorganizational networks (32, 58, 27) - Goal-directed network (52) - Social relations (40) - Social cohesion (38) - Social network (38) - Collaboration (56, 22, 60, 33, 25) - Connections (24, 15) - Relationship with influential others (3) - Quality of human relationships (44) - Linking with others (30) - Networking (51) |
|  | Communication | - Communication (39, 29, 55, 52, 53, 51) - Communication with community members (3) |
| Critical awareness and problem solving |  | - Conflict resolution (39) - Problem assessment (39, 26) - Asking why (22, 23, 25, 30) - Critical reflection (32, 58, 26, 27, 34) - Critical awareness/ reflexivity (33) - Awareness (59) - Community-based solutions (52) - Needs assessment (51) - Problem solving (41) |
| Community structure |  | - Community structures (22, 23, 25) - Organizing (48, 57) - Organizational structures (26) - Organizational development (45) - Policy making (50) - Youth, elders, women (53) - Culture and native language (53) - Community development (29, 43, 31) |
|  | Shared values and goals | - Community values (32, 58, 27) - Value system (40) - Shared vision (23, 52, 56) |
|  | Sustainability | - Sustainability (39, 48, 29) - Personnel sustainability (3) - Continuing activities (51) - Continuing the process (3, 39) |

**Supplementary table 5** Quality assessment of included studies

| **First Author and publication year in alphabetical order** | **Are there clear qualitative and quantitative research questions?** | **Do the collected data allow address the research question?** | **Qualitative studies** | | | | |
| --- | --- | --- | --- | --- | --- | --- | --- |
|  |  |  | Is the qualitative approach appropriate to answer the research question? | Are the qualitative data collection methods adequate to address the research question | Are the findings adequately derived from the data? | Is the interpretation of results sufficiently substantiated by data? | Is there coherence between qualitative data sources, collection, analysis and interpretation? |
| **Baugh-Littlejohns et al. 2000** | ✓ | ✓ | ✓ | ✓ | ✓ | ✓ | ✓ |
| **Garney et al. 2017** | ✓ | ✓ | ✓ | ✓ | ✓ | ✓ | ✓ |
| **Jackson et al. 2003** | ✓ | ✓ | ✓ | ✓ | ✓ | ✓ | ✓ |
| **Jones et al. 2017** | ✓ | ✓ | ✓ | ✓ | ✓ | ✓ | ✓ |
| **Loss et al. 2020** | ✓ | ✓ | ✓ | ✓ | ✓ | ✓ | ✓ |
| **Merzel et al. 2008** | ✓ | ✓ | ✓ | ✓ | ✓ | ✓ | ✓ |
| **Motley et al. 2013** | ✓ | ✓ | ✓ | ✓ | Can’t tell | Can’t tell | Can’t tell |
| **Parker et al. 2010** | ✓ | ✓ | ✓ | ✓ | ✓ | ✓ | ✓ |
| **Postma & Ramon 2016** | ✓ | ✓ | ✓ | ✓ | ✓ | ✓ | ✓ |
| **Raine et al. 2014** | ✓ | ✓ | ✓ | ✓ | ✓ | ✓ | ✓ |
| **Thompson et al. 2000** | ✓ | ✓ | ✓ | ✓ | ✓ | ✓ | ✓ |
| **First Author and publication year in alphabetical order** | **Are there clear qualitative and quantitative research questions?** | **Do the collected data allow address the research question?** | **Quantitative non-randomized** | | | | |
|  |  |  | Are the participants representative of the target population? | Are the participants representative of the target population? | Are there complete outcome data? | Are the confounders accounted for in the design and analysis? | During the study period, is the intervention administered (or exposure occurred) as intended? |
| **Chae et al. 2021** | ✓ | ✓ | ✓ | ✓ | Can’t tell | ✓ | ✓ |
| **Millar et al. 2013** | ✓ | ✓ | ✓ | ✓ | ✓ | ✓ | ✓ |
| **First Author and publication year in alphabetical order** | **Are there clear qualitative and quantitative research questions?** | **Do the collected data allow address the research question?** | **Quantitative descriptive** | | | | |
|  |  |  | Is the sampling strategy relevant to address the research question? | Is the sample representative of the target population? | Are the measurements appropriate? | Is the risk of nonresponse bias low? | Is the statistical analysis appropriate to answer the research question? |
| **Griffin et al. 2005** | ✓ | ✓ | ✓ | Can’t tell | ✓ | Can’t tell | ✓ |
| **Hargreaves et al. 2017** | ✓ | ✓ | ✓ | ✓ | ✓ | ✓ | Can’t tell |
| **Jung et al. 2012** | ✓ | ✓ | ✓ | ✓ | ✓ | ✓ | ✓ |
| **Jung et al. 2013** | ✓ | ✓ | ✓ | ✓ | ✓ | Can‘t tell | ✓ |
| **Kegler et al. 2011** | ✓ | ✓ | ✓ | Can’t tell | ✓ | Can’t tell | ✓ |
| **Nickel et al. 2018** | ✓ | ✓ | ✓ | No | ✓ | Can‘t tell | ✓ |
| **First Author and publication year in alphabetical order** | **Are there clear qualitative and quantitative research questions?** | **Do the collected data allow address the research question?** | **Mixed methods** | | | | |
|  |  |  | Is there an adequate rationale for using a mixed methods design to address the research question? | Are the different components of the study effectively integrated to answer the research question? | Are the outputs of the integration of qualitative and quantitative components adequately interpreted?? | Are divergences and inconsistencies between quantitative and qualitative results adequately addressed? | Do the different components of the study adhere to the quality criteria of each tradition of the methods involved? |
| **Alfonso et al. 2008** | ✓ | ✓ | ✓ | ✓ | Can’t tell | ✓ | Can’t tell |
| **Anderson-Lewis et al. 2012** | ✓ | ✓ | ✓ | ✓ | ✓ | ✓ | ✓ |
| **Atinga et al. 2019** | ✓ | ✓ | ✓ | ✓ | ✓ | ✓ | ✓ |
| **Brazier et al. 2014** | ✓ | ✓ | ✓ | ✓ | ✓ | Can‘t tell | ✓ |
| **Brock et al. 2019** | ✓ | ✓ | ✓ | ✓ | ✓ | ✓ | ✓ |
| **Downey et al. 2010** | ✓ | ✓ | ✓ | ✓ | ✓ | ✓ | Can’t tell |
| **Dressendorfer et al. 2005** | ✓ | ✓ | ✓ | ✓ | Can’t tell | ✓ | Can’t tell |
| **de Groot et al. 2010** | ✓ | ✓ | ✓ | ✓ | ✓ | ✓ | No |
| **Hernantes et al. 2017** | ✓ | ✓ | ✓ | ✓ | ✓ | ✓ | ✓ |
| **Kim et al. 2009** | ✓ | ✓ | ✓ | ✓ | ✓ | ✓ | Can’t tell |
| **Kim et al. 2020** | ✓ | ✓ | ✓ | ✓ | ✓ | Can’t tell | ✓ |
| **Lempa et al. 2008** | ✓ | ✓ | ✓ | ✓ | ✓ | ✓ | ✓ |
| **Lovell et al. 2015, 2017** | ✓ | ✓ | ✓ | ✓ | ✓ | ✓ | ✓ |
| **Maclellan-Wright et al. 2007** | ✓ | ✓ | ✓ | ✓ | ✓ | ✓ | ✓ |
| **Oetzel et al. 2011** | ✓ | ✓ | ✓ | ✓ | ✓ | ✓ | ✓ |
| **Suwanbamrung et al. 2010** | ✓ | ✓ | ✓ | ✓ | ✓ | ✓ | ✓ |
| **Underwood et al. 2012** | ✓ | ✓ | ✓ | ✓ | ✓ | ✓ | ✓ |
| **Van den Broucke et al. 2010** | ✓ | Can‘t tell | ✓ | ✓ | ✓ | ✓ | ✓ |
| **Waqa et al. 2013** | ✓ | ✓ | ✓ | ✓ | ✓ | ✓ | ✓ |

References

1. Mattesich PW, Monsey BR. Collaboration: What Makes It Work. A Review of Research Literature on Factors Influencing Successful Collaboration. Lafond, St. Paul; 1992.

2. Chinman M, Imm P, Wandersman A. Getting To Outcomes: Promoting Accountability Through Methods and Tools for Planning, Implementation, and Evaluation.: Rand Corp; 2004. Available from: URL: https://www.rand.org/pubs/technical_reports/TR101.html.

3. Lempa M, Goodman RM, Rice J, Becker, AB. Development of scales measuring the capacity of community-based initiatives. Health education & behavior 2008; 35(3).

4. Chaskin RJ, Brown P, Venkatesh S, Vidal A. Building Community Capacity. Routledge; 2001.

5. Sandoval JA, Lucero J, Oetzel J, Avila M, Belone L, Mau M et al. Process and outcome constructs for evaluating community-based participatory research projects: a matrix of existing measures. Health Educ Res 2012; 27(4).

6. Public Health Agency of Canada. Community capacity building tool: A tool for planning, building and reflecting on community capacity in community based health projects: Public Health Agency of Canada; 2013 [cited 2022 Mar 10]. Available from: URL: https://www.canada.ca/en/public-health/corporate/mandate/about-agency/regional-operations/community-capacity-building-tool.html.

7. Alfonso ML, Nickelson J, Hogeboom DL, French J, Bryant CA, McDermott RJ et al. Assessing local capacity for health intervention. Eval Program Plann 2008; 31(2):145–59.

8. NSW Health Department. A framework for building capacity to improve health. Sydney, Australia: NSW Health Department; 2001.

9. Bush R, Dower J, Mutch A. Community Capacity Index Manual. Brisbane: Centre for Primary Health Care, the University of Queensland; 2002.

10. Goodman RM, Speers MA, McLeroy KR, Fawcett S, Kegler MC, Parker EA et al. Identifying and defining the dimensions of community capacity to provide a basis for measurement. Health education & behavior 1998; 25(3).

11. Labonte R, Laverack G. Capacity building in health promotion, Part 1: for whom? And for what purpose? Critical Public Health 2001; 11(2).

12. Jackson SF, Cleverly S, Poland B, Burman D, Edwards RW, Robertson A. Working with Toronto neighbourhoods toward developing indicators of community capacity. Health Promot Int 2003; 18(4).

13. Smith N, Littlejohns LB, Roy D. Measuring Community Capacity: State of the Field Review and Recommendations for Future Research; 2003 [cited 2022 Mar 25]. Available from: URL: http://www.dthr.ab.ca/resources/documents/reports/.

14. Wendel ML, Burdine JN, McLeroy KR, Alanziz A, Norton B, Felix MR. Community capacity: theory and application. Emerging Theories in Health Promotion Practice and Research 2009; 302(277).

15. Lovell SA, Gray AR, Boucher SE. Developing and validating a measure of community capacity: Why volunteers make the best neighbours. Soc Sci Med 2015; 133.

16. Sauter A, Lindacher V, Rueter J, Curbach J, Loss J. How Health Promoters Can Assess Capacity Building Processes in Setting-Based Approaches-Development and Testing of a Monitoring Instrument. Int J Environ Res Public Health 2020; 17(2).

17. Oetting ER, Donnermeyer JF, Plested BA, Edwards RW, Kelly K, Beauvais F. Assessing community readiness for prevention. Substance Use Misuse 1995; 30.

18. Gibbon M, Labonte R, Laverack G. Evaluating community capacity. Health Soc Care Community 2002; 10(6):485–91.

19. Norton BL, Burdine JN, Dorsey AM, Felix MJ, McLeroy KR. Community capacity: Concept, theory, and methods. In: DiClemente RJ, Crosby RA, Kegler MC, editors. Emerging theories in health promotion practice and research. San Francisco, CA: Jossey-Bass; 2002.

20. Freudenberg N. Community capacity for environmental health promotion: determinants and implications for practice. Health education & behavior 2004; 31(4):472–90.

21. Bjaras G, Haglund JA, Rifkin SB. A new approach to community participation assessment. Health Promot Int 1991; 6(3).

22. Chae Y, Ha Y. Development and Effectiveness of a Community Capacity Building Program for the Wellness of Traditional Marketplace Merchants: A Pilot Study. Int J Environ Res Public Health 2021; 18(22).

23. Kim J, Koh KW, Yu BC, Jeon MJ, Kim YJ, Kim YH. Assessment of community capacity building ability of health promotion workers in public health centers. Journal of preventive medicine and public health 2009; 42(5).

24. Kim J, Lee H, Cho E, Lee KH, Park CG, Cho B-H. Multilevel Effects of Community Capacity on Active Aging in Community-Dwelling Older Adults in South Korea. Asian Nurs Res (Korean Soc Nurs Sci) 2020; 14(1).

25. MacLellan-Wright M-F, Anderson D, Barber S, Smith N, Cantin B, Felix R et al. The development of measures of community capacity for community-based funding programs in Canada. Health Promot Int 2007; 22(4).

26. Motley M, Holmes A, Hill JL, Plumb K, Zoellner J. Evaluating community capacity to address obesity in the Dan River region: a case study. Am J Health Behav 2013; 37(2).

27. Parker EA, Chung LK, Israel BA, Reü A, Wilkins D. Community organizing network for environmental health: using a community health development approach to increase community capacity around reduction of environmental triggers. J Prim Prev 2010; 31(1-2).

28. Underwood C, Boulay M, Snetro-Plewman G, Macwan'gi M, Vijayaraghavan J, Namfukwe M et al. Community capacity as means to improved health practices and an end in itself: evidence from a multi-stage study. Int Q Community Health Educ 2012; 33(2).

29. Brimblecombe J, van den Boogaard C, Ritchie J, Bailie R, Coveney J, Liberato SC. From targets to ripples: tracing the process of developing a community capacity building appraisal tool with remote Australian Indigenous communities to tackle food security. BMC Public Health 2014; 14.

30. Raine KD, Sosa Hernandez C, Nykiforuk CIJ, Reed S, Montemurro G, Lytvyak E et al. Measuring the progress of capacity building in the Alberta Policy Coalition for Cancer Prevention. Health Promot Pract 2014; 15(4).

31. Thompson D, Smith N. Caught in the web: piloting a methodology to assess community capacity in a rural heart health project [Heart of the Land project]. The Canadian Journal of Program Evaluation 2000; 35(15).

32. Garney WR, Wendel JM, McLeroy KR, Alaniz A, Cunningham G, Castle B et al. Using a Community Health Development Framework to Increase Community Capacity: A Multiple Case Study. Fam Community Health 2017; 40(1):18–23.

33. Loss J, Brew-Sam N, Metz B, Strobl H, Sauter A, Tittlbach S. Capacity Building in Community Stakeholder Groups for Increasing Physical Activity: Results of a Qualitative Study in Two German Communities. Int J Environ Res Public Health 2020; 17(7).

34. Postma J, Ramon C. Strengthening Community Capacity for Environmental Health Promotion through Photovoice. Public Health Nurs 2016; 33(4).

35. Jung M. Covariation in community- and individual-based community capacity and health behavior: a multilevel analysis of populations in Seoul, South Korea. Health Care Manag (Frederick) 2012; 31(4).

36. Lovell SA, Gray AR, Boucher SE. Place, health, and community attachment: Is community capacity associated with self-rated health at the individual level? SSM Popul Health 2017; 3.

37. Brazier E, Fiorentino R, Barry MS, Diallo M. The value of building health promotion capacities within communities: evidence from a maternal health intervention in Guinea. Health Policy Plan 2015; 30(7).

38. Jung M, Rhee HS. Determinants of community capacity influencing residents' health status in Seoul, South Korea. Asia Pac J Public Health 2013; 25(2).

39. Brock D-JP, Estabrooks PA, Hill JL, Barlow ML, Alexander RC, Price BE et al. Building and Sustaining Community Capacity to Address Childhood Obesity: A 3-Year Mixed-Methods Case Study of a Community-Academic Advisory Board. Fam Community Health 2019; 42(1).

40. Jones GJ, Edwards MB, Bocarro JN, Bunds KS, Smith JW. Leveraging community sport organizations to promote community capacity: Strategic outcomes, challenges, and theoretical considerations. Sport Management Review 2018; 21(3). Available from: URL: https://www.sciencedirect.com/science/article/pii/S1441352317300657.

41. van den Broucke S, Jooste H, Tlali M, Moodley V, van Zyl G, Nyamwaya D et al. Strengthening the capacity for health promotion in South Africa through international collaboration. Glob Health Promot 2010; 17(2).

42. Kegler MC, Swan DW. Advancing coalition theory: the effect of coalition factors on community capacity mediated by member engagement. Health Educ Res 2011; 27(4).

43. Downey LH, Castellanos DC, Yadrick K, Threadgill P, Kennedy B, Strickland E et al. Capacity building for health through community-based participatory nutrition intervention research in rural communities. Fam Community Health 2010; 33(3).

44. Baugh-Littlejohns L, GermAnn K, Smith N, Bopp J, Bopp M, Reichel C et al. Integrating Community Capacity Building and Enhanced Primary Health Care Services. Aust J Prim Health 2000; 6(4).

45. de Groot FP, Robertson N, Swinburn BA, de Silva-Sanigorski AM. Increasing community capacity to prevent childhood obesity: challenges, lessons learned and results from the Romp & Chomp intervention. BMC Public Health 2010; 10:522.

46. Millar L, Robertson N, Allender S, Nichols M, Bennett C, Swinburn B. Increasing community capacity and decreasing prevalence of overweight and obesity in a community based intervention among Australian adolescents. Prev Med 2013; 56(6).

47. Atinga RA, Agyepong IA, Esena RK. Willing but unable? Extending theory to investigate community capacity to participate in Ghana's community-based health planning and service implementation. Eval Program Plann 2019; 72.

48. Anderson-Lewis C, Cuy-Castellanos D, Byrd A, Zynda K, Sample A, Blakely Reed V et al. Using mixed methods to measure the perception of community capacity in an academic-community partnership for a walking intervention. Health Promot Pract 2012; 13(6):788–96.

49. Waqa G, Moodie M, Schultz J, Swinburn B. Process evaluation of a community-based intervention program: Healthy Youth Healthy Communities, an adolescent obesity prevention project in Fiji. Glob Health Promot 2013; 20(4).

50. Dressendorfer RH, Raine K, Dyck RJ, Plotnikoff RC, Collins-Nakai RL, McLaughlin WK et al. A conceptual model of community capacity development for health promotion in the Alberta Heart Health Project. Health Promot Pract 2005; 6(1).

51. Suwanbamrung C, Nukan N, Sripon S, Somrongthong R, Singchagchai P. Community capacity for sustainable community-based dengue prevention and control: study of a sub–district in Southern Thailand. Asian Pacific Journal of Tropical Medicine 2010; 3(3). Available from: URL: https://www.sciencedirect.com/science/article/pii/S1995764510600120.

52. Hargreaves MB, Verbitsky-Savitz N, Coffee-Borden B, Perreras L, White CR, Pecora PJ et al. Advancing the measurement of collective community capacity to address adverse childhood experiences and resilience. Children and Youth Services Review 2017; 76. Available from: URL: https://www.sciencedirect.com/science/article/pii/S0190740917301664.

53. Oetzel J, Wallerstein N, Solimon A, Garcia B, Siemon M, Adeky S et al. Creating an instrument to measure people's perception of community capacity in American Indian communities. Health education & behavior 2011; 38(3).

54. Nickel S, Süß W, Lorentz C, Trojan A. Long-term evaluation of community health promotion: using capacity building as an intermediate outcome measure. Public Health 2018; 162.

55. Griffin SF, Reininger BM, Parra-Medina D, Evans AE, Sanderson M, Vincent ML. Development of multidimensional scales to measure key leaders' perceptions of community capacity and organizational capacity for teen pregnancy prevention. Fam Community Health 2005; 28(4):307–19.

56. Brinkman E, Seekamp E, Davenport MA, Brehm JM. Community capacity for watershed conservation: a quantitative assessment of indicators and core dimensions. Environ Manage 2012; 50(4).

57. Goodman RM. A construct for building the capacity of community-based initiatives in racial and ethnic communities: a qualitative cross-case analysis. Journal of public health management and practice 2008; 14 Suppl.

58. Merzel C, Moon-Howard J, Dickerson D, Ramjohn D, VanDevanter N. Making the connections: community capacity for tobacco control in an urban African American community. Am J Community Psychol 2008; 41(1-2):74–88.

59. Hernantes N, Bermejo-Martins E, Øvergård KI, Pumar-Mendez MJ, Lopez-Dicastillo O, Iriarte-Roteta A et al. Theory-based capacity building intervention for intersectoral action for health at local governments: An exploratory pilot study. J Adv Nurs 2022; 78(6).

60. Lee Y-J. Developing a scale of community capacity: testing community organizations in Taiwan. Health Promot Int 2021; 36(6).
