# Supplementary material for: Community capacity for prevention and health promotion: a scoping review on underlying domains and assessment methods
Source: Syst Rev. 2023 Aug 22;12:147. doi: 10.1186/s13643-023-02314-1 (PMC10464111; doi:10.1186/s13643-023-02314-1)
Supplement: Supplementary file 2 — Additional file 2: Appendix 2. Search strategies for each database. [file 13643_2023_2314_MOESM2_ESM.docx]

**Appendix 2**

Search strategies for each database:

MEDLINE / PubMed:

(("community capacity" OR "capacity building" OR "community engagement" OR "community participation" OR "project management") AND (prevention OR "health promotion" OR "community intervention") AND (evaluate*[tiab] OR assess*[tiab] OR measure*[tiab])) AND (English[Lang] AND ("1990/01/01"[PDAT] : "2022/02/06"[PDAT]) NOT letter[pt] NOT comment[pt] NOT editorial[pt] NOT news[pt] NOT congress[pt])

Web of Science:

TS=("community capacity" OR "capacity building" OR "community engagement" OR "community participation" OR "project management") AND (prevention OR "health promotion" OR "community intervention") AND (evaluate* OR assess* OR measure*) AND (Language: (English) AND Timespan: 1990-2022 NOT Document Types: (Editorial Material OR Letter OR Comment OR News Item OR Conference Review))

Science Direct:

TITLE-ABS-KEY ("community capacity" OR "capacity building" OR "community engagement" OR "community participation" OR "project management") AND (prevention OR "health promotion" OR "community intervention") AND (evaluate* OR assess* OR measure*) AND (LANGUAGE (English) AND PUBYEAR > 1989 AND PUBYEAR < 2023 NOT (Editorial OR Letter OR Comment OR News OR Short Survey OR Opinion OR Conference Paper))

Google Scholar:

(("community capacity" OR "capacity building" OR "community engagement" OR "community participation" OR "project management") AND (prevention OR "health promotion" OR "community intervention") AND (evaluate* OR assess* OR measure*)) AND after:1990 AND before:2023 AND (lang:English) -inurl:(Editorial OR Letter OR Comment OR News OR Opinion OR "Conference abstract")
